# Occurrence, Phenotypic and Molecular Characterization of Extended-Spectrum- and AmpC- β-Lactamase Producing Enterobacteriaceae Isolated From Selected Commercial Spinach Supply Chains in South Africa

**DOI:** 10.3389/fmicb.2020.00638

**Published:** 2020-04-15

**Authors:** Loandi Richter, Erika M. du Plessis, Stacey Duvenage, Lise Korsten

**Affiliations:** ^1^Department of Plant and Soil Sciences, University of Pretoria, Pretoria, South Africa; ^2^Department of Science and Technology-National Research Foundation Centre of Excellence in Food Security, Bellville, South Africa

**Keywords:** antimicrobial resistance, ESBL, leafy green vegetables, irrigation water, fresh produce production systems

## Abstract

The increasing occurrence of multidrug-resistant (MDR) extended-spectrum β-lactamase- (ESBL) and/or AmpC β-lactamase-producing Enterobacteriaceae in health care systems, the environment and fresh produce is a serious concern globally. Production practices, processing and subsequent consumption of contaminated raw fruit and vegetables represent a possible human transmission route. The purpose of this study was to determine the presence of ESBL/AmpC-producing Enterobacteriaceae in complete spinach supply chains and to characterize the isolated strains phenotypically (antimicrobial resistance profiles) and genotypically (ESBL/AmpC genetic determinants, detection of class 1, 2, and 3 integrons). Water, soil, fresh produce, and contact surface samples (*n* = 288) from two commercial spinach production systems were screened for ESBL/AmpC-producing Enterobacteriaceae. In total, 14.58% (42/288) of the samples were found to be contaminated after selective enrichment, plating onto chromogenic media and matrix-assisted laser desorption ionization time-of-flight mass spectrometry identity confirmation of presumptive ESBL/AmpC isolates. This included 15.28% (11/72) water and 12.12% (16/132) harvested- and processed spinach, while 25% (15/60) retail spinach samples were found to be contaminated with an increase in isolate abundance and diversity in both scenarios. Dominant species identified included *Serratia fonticola* (45.86%), *Escherichia coli* (20.83%), and *Klebsiella pneumoniae* (18.75%). In total, 48 (81.36%) isolates were phenotypically confirmed as ESBL/AmpC-producing Enterobacteriaceae of which 98% showed a MDR phenotype. Genotypic characterization (PCR of ESBL/AmpC resistance genes and integrons) further revealed the domination of the CTX-M Group 1 ESBL type, followed by TEM and SHV; whilst the CIT-type was the only plasmid-mediated AmpC genetic determinant detected. Integrons were detected in 79.17% (*n* = 38) of the confirmed ESBL/AmpC-producing isolates, of which we highlight the high prevalence of class 3 integrons, detected in 72.92% (*n* = 35) of the isolates, mostly in *S. fonticola*. Class 2 integrons were not detected in this study. This is the first report on the prevalence of ESBL/AmpC-producing Enterobacteriaceae isolated throughout commercial spinach production systems harboring class 1 and/or class 3 integrons in Gauteng Province, South Africa. The results add to the global knowledge base regarding the prevalence and characteristics of ESBL/AmpC-producing Enterobacteriaceae in fresh vegetables and the agricultural environment required for future risk analysis.

## Introduction

The prevalence of multidrug-resistant (MDR) human pathogenic bacteria and their genetic determinants have increased significantly in clinical and environmental settings due to the overuse of antibiotics (Jones-Dias et al., 2016). Subsequently, treatment options for infections become limited, especially when these MDR pathogens harbor genes expressing resistance to extended spectrum antibiotics (Freitag et al., 2018). Production of β-lactamases, including extended-spectrum- and AmpC β-lactamases is one of the most significant resistance mechanisms among Enterobacteriaceae (Östholm, 2014). Enterobacteriaceae is a large family of Gram-negative bacteria present in water, soil, and plants, including fresh vegetables where they form part of the indigenous microbiota (Blaak et al., 2014). The family also includes important foodborne pathogens such as pathogenic *Escherichia coli* and *Salmonella* spp., as well as opportunistic pathogens including *Klebsiella pneumoniae*, *Serratia*- and *Citrobacter* spp. (Baylis et al., 2011).

Extended-spectrum β-lactamase (ESBL) and AmpC β-lactamase enzymes are capable of inactivating nearly all β-lactam antibiotics, differing only in their capacity to hydrolyze fourth-generation cephalosporins (Blaak et al., 2014). The ESBLs are classified as Ambler class A enzymes and include TEM-, SHV-, OXA-, and CTX-M enzymes (Bush and Jacoby, 2010). In the 1980s resistance to third-generation cephalosporins were mainly due to the production of TEM and SHV enzymes (Bush and Jacoby, 2010). However, since the early 2000s, production of CTX-M enzymes have predominantly been reported (Bush and Jacoby, 2010; Ye et al., 2017). AmpC β-lactamases, classified as Ambler class C enzymes, contrast class A enzymes in being active against cephamycins (e.g., cefoxitin) and resistant to inhibition by clavulanic acid (Bush and Jacoby, 2010). Plasmid-mediated AmpC (pAmpC) β-lactamases belong to six families including EBC, CIT, ACC, DHA, FOX and MOX (Jacoby, 2009).

Fresh produce have increasingly been reported to constitute a reservoir of ESBL/AmpC-producing Enterobacteriaceae and their associated genetic determinants (Blaak et al., 2014; Ye et al., 2017; Freitag et al., 2018; Iseppi et al., 2018). Bacteria can readily acquire genes for production of ESBL/AmpC β-lactamases, with mobile genetic elements (e.g., integrons) aiding the dissemination process (Schill et al., 2017). Three classes of integrons, classified based on the more conserved amino acid sequences of the integrase gene (*IntI*), are known to be associated with antimicrobial resistance genes (Machado et al., 2005; Kargar et al., 2014; Deng et al., 2015).

Transfer of MDR ESBL/AmpC-producing Enterobacteriaceae onto fresh produce can occur through the use of contaminated irrigation water or during production via animal manure, during processing, transport, and at the point-of-sale (van Hoek et al., 2015). In fact, contaminated irrigation water has been identified as a main contributor of antimicrobial resistance build up in environmental settings (Soodb et al., 2018). Consumption of contaminated raw vegetables can therefore potentially have a negative impact on human health, as antimicrobial resistance genes can be transferred to commensal bacteria which typically colonize the human gut (Ye et al., 2017). In addition, the WHO has reported that leafy greens in particular represent a higher risk for the consumer (WHO, 2008).

The presence of ESBL/AmpC-producing Enterobacteriaceae on leafy green vegetables at the point of sale have been reported worldwide (Kim et al., 2015; Nüesch-Inderbinen et al., 2015; Zurfluh et al., 2015; Usui et al., 2019). Other studies have evaluated the presence of ESBL-producing Enterobacteriaceae in samples from both retail vegetables and the agricultural environment in Tunisia (Ben Said et al., 2015), China (Ye et al., 2017), and Netherlands (Blaak et al., 2014). In South Africa, transfer of extended spectrum and AmpC β-lactamase genetic determinants between antimicrobial resistant *E. coli* strains from irrigation water to lettuce were reported (Njage and Buys, 2014), while a recent study reported a high prevalence of ESBL/AmpC-producing Enterobacteriaceae on spinach samples at retail (Richter et al., 2019). However, no studies have investigated the spread of ESBL/AmpC-producing Enterobacteriaceae and prevalence of integrons that potentially aid in dispersal of these resistance genes throughout the fresh produce supply chains. This include the on farm environment, harvesting, processing and packaging, up to the point of sale. This study aimed to determine the presence of ESBL/AmpC-producing Enterobacteriaceae in typical commercial spinach production systems from the farm to retail, and to characterize the isolated strains by (i) phenotypic antimicrobial resistance profiles, (ii) identification of ESBL/AmpC genetic determinants, and (iii) detection of Class 1,2 and 3 integrons.

## Materials and Methods

### Sampling Study Areas

Samples were collected from two different commercial spinach production scenarios typically seen in vegetables supply chains in Gauteng Province, SA from June to November 2017. The first scenario consisted of a GLOBAL-GAP certified farm (Farm A) that used river water with overhead irrigation and open field cultivation. Depending on the field layout, river water was either used directly or used after storing in a holding dam. The processing facility was located on the farm where spinach was either washed, dried, cut, packed or made up in bunches, and sent to national fresh produce markets, retailers, and/or retail-distribution centers. The second spinach production scenario used a central processing facility and received produce from various farms. Two GLOBAL-GAP certified farms (Farm B and Farm C, located 112 and 105 km, respectively, from the processing facility) were selected for sampling of baby spinach. Both farms used borehole water for irrigation and produce were grown in tunnels. On Farm B, borehole water was circulated between two holding dams, while one big holding dam was used on Farm C.

### Sample Collection and Processing

A total number of 288 samples were collected throughout the supply chains from the two spinach production scenarios (Supplementary Figure S1). This included soil at harvest (*n* = 6 composite samples); water samples at the source, irrigation point and during processing (*n* = 72); spinach samples at harvest, during processing and at retail (*n* = 192); and contact surface swab samples throughout production and processing of the fresh produce (*n* = 18).

#### Soil

Soil was collected from five replicate points during harvest from the spinach production fields. A composite sample of 25 g (5 g from each replicate) were added to 225 ml buffered peptone water (BPW) and incubated for 3–4 h at 37°C prior to enrichment for detection and isolation of presumptive ESBL/AmpC-producing Enterobacteriaceae.

#### Water

From each water sampling point (source-, irrigation pivot point-, and wash water), 1 L water samples were collected in triplicate and each sample filtered through a 0.45 μm nitrocellulose membrane (Sartorius, Johannesburg, South Africa). The membrane was subsequently placed into 50 ml BPW and incubated for 3–4 h at 37°C prior to enrichment for presumptive ESBL/AmpC-producing Enterobacteriaceae.

#### Fresh Produce

After removal of the spinach stalks, at least three leaves were used to prepare 50 g composite samples. For the baby spinach, 50 g composite samples were obtained. Each sample was aseptically cut and placed into a sterile polyethylene strainer stomacher bag (Seward Ltd., London, United Kingdom) containing 200 ml (3M, Johannesburg) BPW in a 1:4 weight to volume ratio. Individual vegetable samples were blended for 5 min at 230 rpm in a Stomacher 400 circulator paddle blender (Seward Ltd., London) and incubated for 3–4 h at 37°C prior to enrichment for presumptive ESBL/AmpC-producing Enterobacteriaceae.

#### Contact Surfaces

Transystem^TM^ swabs with Amies medium (Lasec, Johannesburg) were used to sample a 25 cm^2^ area from crates, tables, and conveyer belt surfaces, respectively, in triplicate, according to the standard procedures for environmental swab sampling (Public Health England, 2014). Swabs were analyzed by placing each into 9 ml BPW for the 3–4 h enrichment at 37°C prior to enrichment for presumptive ESBL/AmpC-producing Enterobacteriaceae.

### Isolation and Identification of Presumptive ESBL/AmpC-Producing Enterobacteriaceae

Presumptive ESBL/AmpC-producing Enterobacteriaceae were isolated and identified as previously described (Richter et al., 2019). Briefly, each of the prepared BPW-samples were incubated for 3–4 h at 37°C after which 1 ml was added to 9 ml Enterobacteriaceae enrichment (EE) broth (Oxoid, Johannesburg) and incubated overnight at 30°C. Presumptive ESBL/AmpC-producing microorganisms were detected by streaking (10 μl) each of the enriched samples onto ChromID ESBL agar plates (bioMérieux, Midrand, South Africa) and incubated overnight at 30°C (Blaak et al., 2014). All presumptive positive ESBL/AmpC-producing Enterobacteriaceae colonies were isolated and purified. Isolates were identified using matrix assisted laser desorption ionization time-of-flight mass spectrometry (MALDI-TOF) (Bruker, Bremen, Germany) to species level as described by Standing et al. (2013) and AOAC-OMA#2017.09. Briefly, the purified presumptive positive ESBL/AmpC-producing Enterobacteriaceae colonies were regrown in 9 ml tryptone soy broth (TSB) and incubated overnight at 37°C. Subsequently, isolates were streaked out on Nutrient Agar (MERCK) and the plates were incubated overnight at 37°C and subjected to the MALDI Biotyper protocol (Bruker, Bremen, Germany). All strains were tested in duplicate (Supplementary Table S1). The best organism match score values ranging between 2.300 and 3.00 were considered reliable for identification at the species level, whilst the best organism match score values ranging between 2.00 and 2.299 were considered reliable for genus level, with probable species identification, and values between 1.700 and 1.999 were considered as probable genus identification.

### Antimicrobial Susceptibility Testing

Antimicrobial susceptibility was tested using the Kirby Bauer disk diffusion technique (CLSI, 2018). All isolates were screened for ESBL production by the double-disk synergy test (DDST) using cefotaxime-30 μg, ceftazidime-30 μg, and cefpodoxime-10 μg, alone or in combination with clavulanic acid-10 μg (Mast Diagnostics, Randburg, South Africa) (EUCAST, 2013). To determine if isolates were resistant, intermediate, or susceptible, zone diameters were measured and compared to the CLSI and EUCAST criteria. Isolates showing resistance to cefoxitin and cefotaxime or ceftazidime were regarded as a phenotypic indicator of AmpC production (EUCAST, 2013). The cefepime ESBL disc set (Cefepime-30 μg, cefepime-clavulanic acid-30 μg-10 μg) and the AmpC detection set (Mast Diagnostics, Randburg) were used to confirm ESBL and AmpC production, respectively (EUCAST, 2013; CLSI, 2018). Resistance or susceptibility of isolates were also tested using ampicillin-10 μg, augmentin-20 μg/10 μg, amoxicillin-10 μg, cotrimoxazole-1.25 μg/23.75 μg, imipenem-10 μg, neomycin-10 μg, tetracycline-30 μg, gentamycin-10 μg, chloramphenicol-10 μg (Mast Diagnostics) (CLSI, 2018). Isolates resistant to three or more antimicrobial classes were regarded MDR. According to the manufacturers’ instructions *K. pneumoniae* ATCC 700603, *E. coli* NCTC 13351, and *Enterobacter cloacae* NCTC 1406 were used as positive controls and *E. coli* ATCC 25922 were included as a negative control (Mast Diagnostics).

### Detection of β-Lactamase Genes and Integrons

All confirmed ESBL/AmpC-producing isolates were analyzed by PCR and sequencing for the presence of ESBL determinants (*bla*TEM, *bla*SHV, *bla*CTX-M, *bla*OXA) and plasmid-mediated AmpC (pAmpC) resistance genes (*bla*ACC, *bla*FOX, *bla*MOX, *bla*DHA, *bla*CIT, *bla*EBC) as well as class 1, 2, and 3 integrons (*IntI*1, *IntI*2, *IntI*3). Single colonies of each isolate were cultured aerobically under shaking conditions at 200 rpm in TSB (MERCK, Johannesburg) for 24 h at 30°C. The cells were pelleted by centrifugation (12,500 *g* for 10 min), DNA was extracted using the Quick-gDNA Mini-Prep kit (Zymo Research, Irvine, CA, United States) and the DNA concentration was determined using the Qubit dsDNA Broad Range Assay and a Qubit 2.0 fluorometer (Life Technologies, Johannesburg). PCR was performed using the DreamTaq Green PCR Master Mix (Thermo Fisher Scientific, Johannesburg) with specific primers and thermocycling conditions for each of the genes as described in Table 1. PCR products were sequenced using BigDye Terminator v3.1 cycle sequencing on an ABI 3500XL sequencer in forward and reverse direction (Inqaba Biotec, Johannesburg). The sequences were edited with Chromas 2.6 and BioEdit sequence alignment editor software and consensus sequences were subjected to BLAST nucleotide search analysis to identify the antimicrobial resistance genes.

**TABLE 1:** Primers used for screening of broad-spectrum β-lactamase, ESBL, and AmpC genetic determinants (Dallenne et al., 2010) as well as integron prevalence (de Paula et al., 2018) in selected Enterobacteriaceae isolated from water, fresh produce, and contact surfaces.

Target genes	Primer sequences	Thermocycling conditions	Expected amplicon size (bp)
*bla*_*TEM*_	TEM-F: 5′-CATTTCCGTGTCGCCCTTATTC-3′	94°C, 10 min;	800
	TEM-R: 5′-CGTTCATCCATAGTTGCCTGAC-3′	30 cycles of	
*bla*_*SHV*_	SHV-F: 5′-AGCCGCTTGAGCAAATTAAAC-3′	94°C, 40 s,	713
	SHV-R: 5′-ATCCCGCAGATAAATCACCAC-3′	58°C, 40 s,	
*bla*_*OXA–*__1__like_	OXA-F: 5′-GGCACCAGATTCAACTTCAAG-3′	72°C, 1 min;	564
	OXA-R: 5′-GACCCCAAGTTTCCTGTAAGTG-3′	72°C, 7min	

*bla*_*CTX–M Group*8/25_	CTX-M Gp8/25-F: 5′-AACRCRCAGACGCTCTAC-3′	94°C, 10 min;	326
	CTX-M Gp8/25-R: 5′-TCGAGCCGGAASGTGTYAT-3′	30 cycles of	
*bla*_*CTX–M Group* 9_	CTX-M Gp9-F: 5′-TCAAGCCTGCCGATCTGGT-3′	94°C, 40 s,	688
	CTX-M Gp9-R: 5′-TGATTCTCGCCGCTGAAG-3′	60°C, 40 s,	
*bla*_*CTX–M Group* 1_	CTX-M Gp1-F: 5′-TTAGGAARTGTGCCGCTGYA-3′	72°C, 1 min;	561
	CTX-M Gp1-R: 5′-CGATATCGTTGGTGGTRCCAT-3′	72°C, 7 min	

*bla*_*ACC*_	ACC-F: 5′-CACCTCCAGCGACTTGTTAC-3′ ACC-R: 5′-GTTAGCCAGCATCACGATCC-3′	94°C, 10 min;	346
		30 cycles of	
		94°C, 40 s,	
		60.5°C, 40 s,	
		72°C, 1 min;	
		72°C, 7 min	

*bla*_*FOX*_	FOX-F: 5′-CTACAGTGCGGGTGGTTT-3′	94°C, 10 min;	162
	FOX-R: 5′-CTATTTGCGGCCAGGTGA-3′	30 cycles of	
*bla*_*MOX*_	MOX-F: 5′-GCAACAACGACAATCCATCCT-3′	94°C, 40 s,	895
	MOX-R: 5′-GGGATAGGCGTAACTCTCCCAA-3′	59.6°C, 40 s,	
*bla*_*DHA*_	DHA-F: 5′-TGATGGCACAGCAGGATATTC-3′	72°C, 1 min;	997
	DHA-R: 5′-GCTTTGACTCTTTCGGTATTCG-3′	72°C, 7 min	
*bla*_*CIT*_	CIT-F: 5′-CGAAGAGGCAATGACCAGAC-3′		538
	CIT-R: 5′-ACGGACAGGGTTAGGATAGY-3′		
*bla*_*EBC*_	EBC-F: 5′-CGGTAAAGCCGATGTTGCG-3′		683
	EBC-R: 5′-AGCCTAACCCCTGATACA-3′		

*IntI1*	Int1-F: 5′-GGT CAAGGATCTGGATTTCG-3′	94°C, 12 min;	436
	Int1-R: 5′-ACATGCGTGTAAATCATCGTC-3′	30 cycles of	
*IntI2*	Int2-F: 5′-CACGGATATGCGACAAAAAGG-3′	94°C, 30 s,	788
	Int2-R: 5′-TGTAGCAAACGAGTGACGAAATG-3′	60°C, 30s,	
*IntI3*	Int3-F: 5′-AGTGGGTGGCGAATGAGTG-3′	72°C, 1 min;	600
	Int3-R: 5′-TGTTCTTGTATCGGCAGGTG-3′	72°C, 8 min	

## Results

### Isolation and Identification of Presumptive ESBL/AmpC-Producing Enterobacteriaceae Isolates

Presumptive ESBL/AmpC-producing Enterobacteriaceae (*n* = 59) from the selective chromogenic media belonged to six genera including *Escherichia*, *Klebsiella*, *Serratia*, *Rahnella*, *Salmonella*, and *Enterobacter*, with MALDI-TOF analysis (Supplementary Table S1). All presumptive ESBL/AmpC-producing Enterobacteriaceae from the selective chromogenic media had best organism match score values >1.700 and <3.00 (Supplementary Table S1). According to the MALDI-TOF score value description, a total of 66.10% of the isolates were characterized to highly probable species identification, 27.12% were characterized to secure genus identification and probable species identification, whilst 6.78% were characterized to probable genus identification (Supplementary Table S1). This included isolates from the water (*n* = 20), fresh produce (*n* = 35) and contact surface samples (*n* = 4), while no presumptive ESBL/AmpC-producing Enterobacteriaceae isolates were recovered from the soil samples.

### Prevalence of ESBL/AmpC-Producing Enterobacteriaceae and Antimicrobial Susceptibility Testing

**FIGURE 1:**
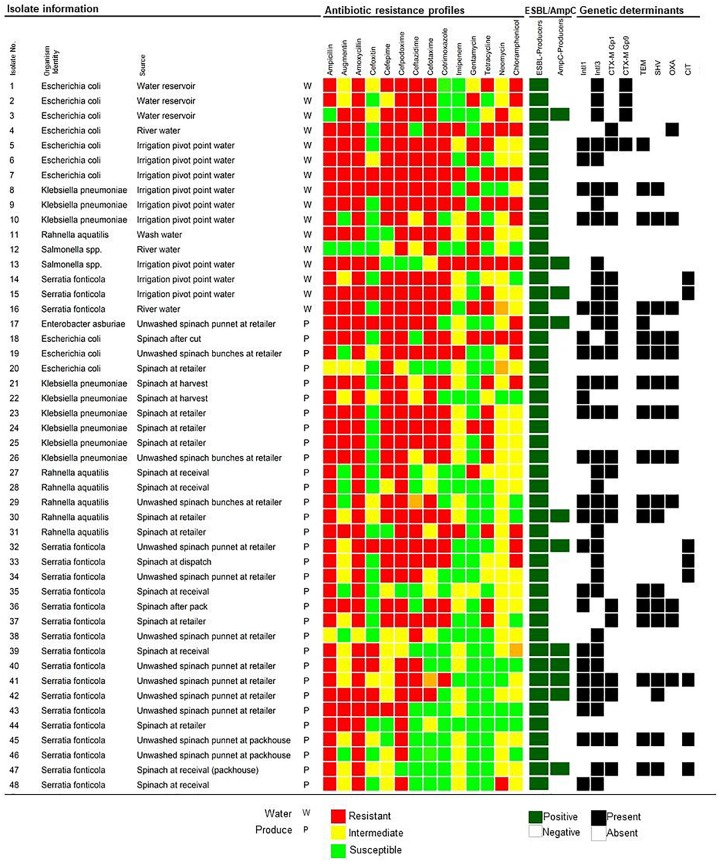
Extended-Spectrum- and AmpC- β-Lactamase producing Enterobacteriaceae isolated from water, spinach, and contact surface sources, indicating the phenotypic antibiotic resistance profiles and the detection of ESBL and/or AmpC, and integron genetic determinants. The color code of the antimicrobial resistance profiles indicate the resistant, intermediate resistant, or susceptible phenotypes to specific antibiotics from seven different classes. ESBL/AmpC production is indicated as positive or negative and detection of genetic determinants indicated as present or absent.

In total, screening using DDST, 48/59 (81.36%) isolates tested positive for ESBL production (Figure 1). All cefoxitin resistant isolates (20/59) were additionally screened with the AmpC detection set of which 11/20 (55%) tested positive (Figure 1). From the 48 ESBL/AmpC-producing isolates, 16 isolates were from water and 32 from produce samples. Irrigation water isolates (*n* = 15) included *E. coli* (14.58%) and *Serratia fonticola* (6.25%) from both scenarios, while *K. pneumoniae* (6.25%) and *Salmonella* spp. (4.17%) were isolated only from scenario 1 where river water was used for irrigation. Isolates from the spinach at harvest and throughout processing (*n* = 13) included predominantly *S. fonticola* (16.67%), followed by *K. pneumoniae* (4.17%), *Rahnella aquatilis* (4.17%) and *E. coli* (2.08%). From the retailer spinach (*n* = 19), ESBL/AmpC-producing *S. fonticola* (16.67%), *K. pneumoniae* (8.33%), *R. aquatilis* (6.25%), *E. coli* (4.17%), and *Enterobacter asburiae* (2.08%) were recovered. One *R. aquatilis* isolate was also recovered from the wash water used during processing in scenario 1 (Figure 1).

Multidrug resistance was observed in 98% of the confirmed ESBL/AmpC-producing isolates, including 16 and 31 isolates from water and fresh produce, respectively (Figure 1). Resistance to the aminoglycoside (89.58%) and chloramphenicol (79.17%) classes were dominant.

Within the β-lactam group, further analysis showed resistance against amoxicillin (31.25% in water and 66.67% in produce), followed by ampicillin (29.17% in water and 66.67% in produce), augmentin (29.17% in water and 52.08% in produce), and cefoxitin (14.58% in water and 27.08% in produce). The resistance rate to carbapenems (imipenem) were 8.33 and 4.17% in water and produce, respectively, with 10.42 and 41.67% of the water and produce isolates that showed intermediate resistance to imipenem. Resistance to other antibiotics included cotrimoxazole (22.92% in water and 29.17% in produce) and tetracycline (22.92% in water and 27.08% in produce).

### Genotypic Antibiotic Resistance Profiling

Genes encoding β-lactamases were detected in 29/48 (60.42%) isolates obtained from water and produce samples, mainly in *S. fonticola* (*n* = 13), followed by *E. coli* (*n* = 7), and *K. pneumoniae* (*n* = 5). The most frequently detected β-lactamase genes were *bla*CTX-M (*n* = 25), followed by *bla*TEM (*n* = 18), *bla*_*SHV*_ (*n* = 17), and *bla*OXA (*n* = 12). ESBL variants encoded by *bla*CTX-M Group 1 included CTX-M-3, CTX-M-12, and CTX-M-15 amongst others, whilst *bla*CTX-M Group 9 encoded for CTX-M-14. The *bla*_*TEM*_ sequences were found to encode for the broad-spectrum β-lactamase TEM-1 and TEM-234. The *bla*_*SHV*_ sequences encoded SHV-187, SHV-203, or SHV-61. All the *bla*OXA sequences encoded broad-spectrum β-lactamases OXA-1. Only the CIT family (identified as *bla*_*CMY*_ variants) of AmpC genetic determinants was detected in six *S. fonticola* isolates from scenario 2 (Figure 1).

### Detection of Integrons

The integrase 1 gene (*IntI1*) was detected in 23/48 (47.92%) of the isolates, predominantly in *S. fonticola* (*n* =11), followed by *K. pneumoniae* (*n* =6), *R. aquatilis* (*n* =2), *E. coli* (*n* = 3), and one *E. asburiae* isolate. The *IntI3* gene associated with class 3 integrons were detected in 35/48 (72.92%) of the isolates, including *S. fonticola* (*n* =16), six *E. coli*, six *K. pneumoniae*, five *R. aquatilis*, and one *E. asburiae* and *Salmonella* spp. isolate, respectively. Both the class 1 and class 3 integrase genes were detected in 29 isolates, which included *S. fonticola* (*n* = 9), *K. pneumoniae* (*n* = 5), *E. coli* (*n* = 3), *R. aquatilis* (*n* = 2), and *E. asburiae* (*n* = 1). Class 2 integrons were not detected in any of the isolates (Figure 1).

## Discussion

This study documents the prevalence of ESBL/AmpC-producing Enterobacteriaceae in spinach production, from the agricultural environment, during processing, and subsequent retailed products in South Africa. Overall, six ESBL/AmpC-producing Enterobacteriaceae genera, including environmental bacteria (*S. fonticola* and *R. aquatilis*), and potential human pathogens (*E. coli, K. pneumoniae*, *Salmonella* spp., and *E*. *asburiae*) were detected from 42 of the 288 samples. From the first production scenario, ESBL-producing potential pathogenic Enterobacteriaceae were mainly isolated, whereas the predominance of ESBL-producing *S. fonticola* from the second production scenario correspond to environmental ESBL-producing Enterobacteriaceae previously reported (Blaak et al., 2014).

Irrigation water is a known source of antimicrobial resistant bacterial contamination in fresh produce production (Vital et al., 2018). In both spinach production scenarios, the prevalence of ESBL/AmpC-producing Enterobacteriaceae (*n* = 48) was higher in samples from produce (29.17 and 37.5%, respectively) than river (20.83%), and borehole (10.42%) water. Similarly, Njage and Buys (2014) reported highest prevalence of ESBL-producing *E. coli* isolates in fresh produce (lettuce) at harvest (90%), followed by different irrigation water (canal, 73% and river, 64%) samples in South Africa. In contrast, 100% irrigation water samples and only 14.7% of the harvested lettuce samples were found to be positive for ESBL/AmpC-producing environmental Enterobacteriaceae in Netherlands (Blaak et al., 2014). The 20.83% (10/48) occurrence of ESBL/AmpC-producing isolates from river irrigation water was higher than the 13.2% reported in a similar study from river water in China (Ye et al., 2017). Potential pathogenic ESBL-producing *K. pneumoniae*, *E. coli*, and *Salmonella* spp. found in our river water samples were similar to the ESBL-producing potential pathogenic *E. coli*, *Citrobacter freundii*, and *K. pneumoniae* reported by Ye et al. (2017). In contrast to Zekar et al. (2017), a 10.4% occurrence of ESBL/AmpC-producing isolates (*E. coli* and *S. fonticola*) was found in borehole irrigation water from the second production scenario. The occurrence of ESBL/AmpC-producing Enterobacteriaceae on all our spinach samples increased from 6.25% at harvest, to 34.38% after processing, up to 59.36% in retail spinach samples in both production scenarios. Furthermore, an increase in species diversity from harvested, to processed-, and subsequent retail spinach were also observed. The identified species on retailer spinach samples included ESBL/AmpC-producing *K. pneumoniae*, *S. fonticola*, *R. aquatilis*, *E. coli*, and *E. asburiae*, similar to other studies (Ye et al., 2017; Zekar et al., 2017; Richter et al., 2019). Interestingly, no ESBL/AmpC-producing Enterobacteriaceae isolates were detected in soil samples from any of the farms analyzed in the current study, which contrasts to Blaak et al. (2014) and Ben Said et al. (2015), where ESBL/AmpC-producing *E. coli* and *S. fonticola* respectively, were detected in soil samples at harvest, respectively.

In this study, 98% of the ESBL/AmpC-producing isolates were multidrug resistant, while 93.3% MDR have been reported for ESBL-producing isolates from a similar study in Tunisia (Ben Said et al., 2015). Moreover, 100% of the river irrigation water isolates from this study showed MDR phenotypes, which is significantly higher than the 42.3% MDR previously reported in ESBL-producing Enterobacteriaceae isolates from river water (Ye et al., 2017). Overall, 63.16% (12/19) of the isolates from retailed spinach showed a MDR phenotype, which is lower than the 83.78% MDR previously reported on retail spinach in South Africa (Richter et al., 2019). In addition, resistance to as many as four additional non-β-lactam antibiotic classes were observed in the MDR ESBL-producing potential pathogenic isolates from river water and spinach samples. This included *K. pneumoniae* isolates with resistance to cotrimoxazole, a clinically relevant antibiotic, similar to clinical isolates in a recent South African study (Vasaikar et al., 2017). The occurrence (36%) of MDR ESBL-producing *K. pneumoniae* throughout the first production scenario was high, compared to similar studies where 0% (Netherlands) and 15% (China) occurrence have been reported (Blaak et al., 2014; Ye et al., 2017). This highlights the potential role that the agricultural environment may have as a reservoir of MDR opportunistic pathogens in fresh produce production. However, the importance of not only assessing the agricultural environment as a possible source of antimicrobial contamination in fresh produce, but also the processing and distribution steps were discussed in a recent review (Hölzel et al., 2018). Accordingly, all ESBL-producing isolates from spinach (*n* = 18) in the second production scenario of this study were isolated from produce during processing and retail (distribution), of which 94.4% showed a MDR phenotype. Interestingly, from the supplier farm where no isolates were found in the agricultural environment, resistance against a maximum of one additional non-β-lactam antibiotic class was seen in the MDR ESBL-producing environmental strains, contrasting the majority of resistance profiles from the other supply chains in this study.

Molecular characterization of the MDR ESBL/AmpC-producing Enterobacteriaceae isolates from both spinach production scenarios revealed the dominance of *bla*_*CTX–M*_, followed by *bla*_*SHV*_ and *bla*_*TEM*_. Worldwide SHV, TEM and CTX-M β-lactamases are the major ESBLs detected in clinical and agricultural settings, including fresh produce (Njage and Buys, 2014; Zhang et al., 2015, Ye et al., 2017). The most common variants reported in literature to date include *bla*_*CTX–M–*__14_ (CTX-M Group 9) and *bla*_*CTX–M–*__15_ (CTX-M Group 1). In our study, CTX-M group 9 (*bla*_*CTX–M–*__14_) was found in *E. coli* isolates from river irrigation water as well as the holding dam borehole water. This corresponds to *E. coli* isolates from river water reported by Njage and Buys (2014). Interestingly, for the CTX-M Group 1 ESBLs detected in our study, variants found in the first processing scenario included *bla*_*CTX–M–*__1_ and *bla*_*CTX–M–*__15_ from *E. coli*, *K. pneumoniae*, and *S. fonticola* isolated from river, irrigation pivot point water, harvested- and retailed spinach samples, whilst in the second processing scenario, CTX-M Group 1 variants included *bla*_*CTX–M–*__3_, *bla*_*CTX–M–*__206_, and *bla*_*CTX–M–*__12_ from *S. fonticola* and *E. asburiae* isolated from spinach samples during processing and at retail. Previous studies have reported *bla*_*CTX–M–*__14_ and *bla*_*CTX–M–*__15_ as the most broadly dispersed in clinical isolates, whilst in environmental isolates, CTX-M Group 1 variants (*bla*_*CTX–M–*__1_ and *bla*_*CTX–M–*__3_ among other), have been reported (Cantón et al., 2012; Borgogna et al., 2016). Additionally, CTX-M Group 1 variants (*bla*_*CTX–M–*__15_, *bla*_*CTX–M–*__3_, and *bla*_*CTX–M–*__12_) found in the different Enterobacteriaceae isolates from vegetables corresponded to other studies (Ye et al., 2017; Richter et al., 2019). Apart from the ESBL genes, pAmpC resistance genes were also detected in six *S. fonticola* isolates from the second production scenario, but only included the CIT type (identified as *bla*_*CMY*_ variants). This is in contrast to our previous findings in produce at the point of sale where the EBC type was predominantly detected from different Enterobacteriaceae species (Richter et al., 2019), but corresponds to a study by Njage and Buys (2014), who predominantly detected the CIT type pAmpC β-lactamases in *E. coli* isolated from lettuce and irrigation water samples in the North West Province, SA.

A high percentage of the ESBL/AmpC-producing isolates in the current study further harbored integrons, which is consistent with previous reports (Ben Said et al., 2015; Ye et al., 2017). Class 1 integrons were detected in 47.96% of the MDR ESBL/AmpC-producing isolates from both scenarios, corresponding to results reported (Ma et al., 2017; Ye et al., 2017). Similar to results reported by Freitag et al. (2018), no class 2 integrons were detected in the current study. This contrasts to previous studies where class 2 integrons were predominantly detected, followed by class 1 integrons from raw salad vegetables retailed in Canada (Bezanson et al., 2008). In this study it was interesting that class 3 integrons were the most prevalent, detected in 72.92% (35/48) ESBL/AmpC-producing isolates. This contrasts previous studies where only class 1 integrons were detected from water and retail food samples (Ye et al., 2017). Co-existence of *IntI1* and *IntI3* was determined in 41.67% (20/48) of the environmental and potential pathogenic isolates from water and spinach samples in production scenario 1 and S. fonticola isolates from processed and retail spinach in production scenario 2, which is a higher occurrence than the 2.9% reported by Kargar et al. (2014) in *E. coli* isolates from a clinical setting. To the best of our knowledge, the only report of class 3 integron detection from vegetables was in a *K. pneumoniae* isolate (Jones-Dias et al., 2016). Identification of class 3 integrons have further been associated with less than ten Enterobacteriaceae genera in isolates of environmental (*Enterobacter* and *Delftia*) and clinical (*Serratia*, *Klebsiella*, and *Escherichia*) origin (Barraud et al., 2013; Jones-Dias et al., 2016; Rajkumari et al., 2018). In our study, class 3 integrons were predominantly detected in the environmental *S. fonticola* isolates throughout each of the supply chains. Future studies will include characterization of these integrons for determination of the gene cassettes encoding specific resistance genes present and the potential role that this class of integrons and ESBL/AmpC-producing environmental Enterobacteriaceae have in the spread of resistance genes in the agroecosystem.

## Conclusion

This is the first study to show the presence of ESBL/AmpC-producing Enterobacteriaceae in the agricultural environment, throughout processing, and the retailer spinach samples. Where river water was used for irrigation, higher contamination levels were seen in the fresh produce supply chains, including an increase in ESBL/AmpC-producing Enterobacteriaceae genera isolated, as well as the phenotypic multidrug resistance profiles. This highlights the importance of the microbiological quality of irrigation water used for fresh produce to be eaten raw. Furthermore, in both spinach production scenarios, the abundance and diversity of ESBL/AmpC-producing Enterobacteriaceae on retailer spinach samples increased. This study therefore showed that Enterobacteriaceae with expanded spectrum antimicrobial resistance are prevalent in selected fresh produce supply chains and moreover, that the resistance genes persist, with ESBL/AmpC-producing MDR organisms remaining present on fresh produce throughout processing in different production systems. The prevalence of MDR ESBL/AmpC-producing Enterobacteriaceae harboring class 1 and class 3 integrons throughout complete spinach production systems highlights the importance of further surveillance of antimicrobial resistance in different environmental settings. In addition, this study adds to the global knowledge base regarding the prevalence and characteristics of ESBL/AmpC-producing Enterobacteriaceae in fresh vegetables and the agricultural environment required for future risk analysis.

## Data Availability Statement

The raw data supporting the conclusions of this article will be made available by the authors, without undue reservation, to any qualified researcher.

## Author Contributions

EP, SD, and LK contributed to the conception of the study. LR, EP, and SD contributed to the design. LR and EP proceeded to identify fresh produce supply chain sampling sites, sample collection, preparation, bacterial isolation, laboratory experiments, and analysis. EP and SD guided and contributed to results analysis, interpretation, and presentation. All authors contributed to manuscript writing, and approved the submitted version.

## Conflict of Interest

The authors declare that the research was conducted in the absence of any commercial or financial relationships that could be construed as a potential conflict of interest.
