# Supplementary material for: Occurrence, Phenotypic and Molecular Characterization of Extended-Spectrum- and AmpC- β-Lactamase Producing Enterobacteriaceae Isolated From Selected Commercial Spinach Supply Chains in South Africa
Source: Front Microbiol. 2020 Apr 15;11:638. doi: 10.3389/fmicb.2020.00638 (PMC7176360; doi:10.3389/fmicb.2020.00638)
Supplement: Supplementary file 1 [file Data_Sheet_1.docx]

Supplementary Table S1: Score values of identified Enterobacteriaceae presumed to be extended-spectrum β-lactamase and/or AmpC-producing strains, isolated from two fresh produce production systems

| **Isolate number** | **Source** | **Organism identity** | **Organism best match*** | | **Range**** | **Consistency category***** |
| --- | --- | --- | --- | --- | --- | --- |
|  |  |  | **Score 1** | **Score 2** |  |  |
| 1 | Water reservoir | *Escherichia coli* | 2,495 | 2,46 | +++ | A |
| 2 | Water reservoir | *Escherichia coli* | 2,47 | 2,383 | +++ | A |
| 3 | Water reservoir | *Escherichia coli* | 2,433 | 2,365 | +++ | A |
| 4 | River water | *Escherichia coli* | 2,475 | 2,443 | +++ | A |
| 5 | Irrigation pivot point water | *Escherichia coli* | 2,397 | 2,458 | +++ | A |
| 6 | Irrigation pivot point water | *Escherichia coli* | 2,375 | 2,359 | +++ | A |
| 7 | Irrigation pivot point water | *Escherichia coli* | 2,388 | 2,533 | +++ | A |
| 8 | Irrigation pivot point water | *Klebsiella pneumoniae* | 2,21 | 2,4 | ++ | A |
| 9 | Irrigation pivot point water | *Klebsiella pneumoniae* | 2,364 | 2,43 | +++ | A |
| 10 | Irrigation pivot point water | *Klebsiella pneumoniae* | 2,435 | 2,435 | +++ | A |
| 11 | Wash water | *Rahnella aquatilis* | 1,981 | 1,984 | + | C |
| 12 | River water | *Salmonella spp.* | 2,227 | 2,397 | +++ | A |
| 13 | Irrigation pivot point water | *Salmonella spp.* | 2,037 | 2,271 | ++ | A |
| 14 | Irrigation pivot point water | *Serratia fonticola* | 2,346 | 2,381 | +++ | A |
| 15 | Irrigation pivot point water | *Serratia fonticola* | 2,422 | 2,488 | +++ | A |
| 16 | River water | *Serratia fonticola* | 2,234 | 2,365 | ++ | A |
| 17 | Unwashed spinach punnet at retailer | *Enterobacter asburiae* | 1,873 | 2,001 | ++ | C |
| 18 | Spinach after cut | *Escherichia coli* | 2,464 | 2,407 | +++ | A |
| 19 | Unwashed spinach bunches at retailer | *Escherichia coli* | 2,429 | 2,51 | +++ | A |
| 20 | Spinach at retailer | *Escherichia coli* | 2,476 | 2,552 | +++ | A |
| 21 | Spinach at harvest | *Klebsiella pneumoniae* | 2,424 | 2,498 | +++ | A |
| 22 | Spinach at harvest | *Klebsiella pneumoniae* | 2,49 | 2,523 | +++ | A |
| 23 | Spinach at retailer | *Klebsiella pneumoniae* | 2,465 | 2,522 | +++ | A |
| 24 | Spinach at retailer | *Klebsiella pneumoniae* | 2,445 | 2,504 | +++ | A |
| 25 | Spinach at retailer | *Klebsiella pneumoniae* | 2,512 | 2,503 | +++ | A |
| 26 | Unwashed spinach bunches at retailer | *Klebsiella pneumoniae* | 2,493 | 2,548 | +++ | A |
| 27 | Spinach at receival | *Rahnella aquatilis* | 2,055 | 2,073 | ++ | C |
| 28 | Spinach at receival | *Rahnella aquatilis* | 2,334 | 2,396 | +++ | C |
| 29 | Unwashed spinach bunches at retailer | *Rahnella aquatilis* | 1,97 | 2,044 | + | B |
| 30 | Spinach at retailer | *Rahnella aquatilis* | 1,91 | 1,962 | + | C |
| 31 | Spinach at retailer | *Rahnella aquatilis* | 1,917 | 1,931 | + | B |
| 32 | Unwashed spinach punnet at retailer | *Serratia fonticola* | 2,074 | 2,42 | +++ | A |
| 33 | Spinach at dispatch | *Serratia fonticola* | 2,267 | 2,399 | +++ | A |
| 34 | Unwashed spinach punnet at retailer | *Serratia fonticola* | 2,191 | 2,294 | ++ | A |
| 35 | Spinach at receival | *Serratia fonticola* | 2,225 | 2,245 | ++ | A |
| 36 | Spinach after pack | *Serratia fonticola* | 2,347 | 2,344 | +++ | C |
| 37 | Spinach at retailer | *Serratia fonticola* | 2,416 | 2,411 | +++ | A |
| 38 | Unwashed spinach punnet at retailer | *Serratia fonticola* | 2,189 | 2,255 | ++ | A |
| 39 | Spinach at receival | *Serratia fonticola* | 2,336 | 2,418 | +++ | A |

Supplementary Table S1 continued

| **Isolate number** | **Source** | **Organism identity** | **Organism best match*** | | **Range**** | **Consistency category***** |
| --- | --- | --- | --- | --- | --- | --- |
|  |  |  | **Score 1** | **Score 2** |  |  |
| 40 | Unwashed spinach punnet at retailer | *Serratia fonticola* | 2,423 | 2,378 | +++ | A |
| 41 | Unwashed spinach punnet at retailer | *Serratia fonticola* | 2,406 | 2,431 | +++ | A |
| 42 | Unwashed spinach punnet at retailer | *Serratia fonticola* | 2,336 | 2,357 | +++ | A |
| 43 | Unwashed spinach punnet at retailer | *Serratia fonticola* | 2,305 | 2,342 | +++ | A |
|  |  |  |  |  |  |  |
|  |  |  |  |  |  |  |
| 44 | Spinach at retailer | *Serratia fonticola* | 2,382 | 2,364 | +++ | A |
| 45 | Unwashed spinach punnet at packhouse | *Serratia fonticola* | 2,241 | 2,202 | ++ | A |
| 46 | Unwashed spinach punnet at packhouse | *Serratia fonticola* | 2,338 | 2,425 | +++ | A |
| 47 | Spinach at receival (packhouse) | *Serratia fonticola* | 2,343 | 2,299 | ++ | A |
| 48 | Spinach at receival | *Serratia fonticola* | 2,476 | 2,347 | +++ | A |
| 49 | Contact surfaces (packhouse) | *Serratia fonticola* | 2,32 | 2,387 | +++ | A |
| 50 | Contact surfaces (packhouse) | *Serratia fonticola* | 2,338 | 2,375 | +++ | C |
| 51 | Unwashed spinach at dispatch (packhouse) | *Escherichia coli* | 2,525 | 2,523 | +++ | A |
| 52 | Holding dam water (source water) | *Escherichia coli* | 2,446 | 2,63 | +++ | A |
| 53 | Contact surfaces (packhouse) | *Rahnella aquatilis* | 2,405 | 2,436 | +++ | C |
| 54 | River water | *Salmonella spp.* | 2,302 | 2,282 | ++ | A |
| 55 | Irrigation pivot point water | *Salmonella spp.* | 2,19 | 2,147 | ++ | C |
| 56 | Spinach at harvest | *Serratia fonticola* | 2,06 | 2,206 | ++ | A |
| 57 | Unwashed spinach punnet at retailer | *Serratia fonticola* | 2,205 | 2,349 | ++ | A |
| 58 | Spinach at receival (packhouse) | *Serratia fonticola* | 2,155 | 2,292 | ++ | A |
| 59 | Contact surfaces (packhouse) | *Serratia fonticola* | 2,194 | 2,314 | ++ | A |
| Control strain: Bruker Bacterial Test Standard (BTS): consists of a manufactured extract of *Escherichia coli* DH5 alpha. Isolates 1 – 48 were subsequently confirmed as ESBL/AmpC-producing Enterobacteriaceae. * All isolates were measured in duplicate; ** Range description: 2.300- 3.000 (+++) highly probable species identification; 2.000- 2.299 (++) secure genus identification, probable species identification; 1.700- 1.999 (+) probable genus identification; 0.000- 1.699 (-) not reliable identification; *** Consistency categories description: (A) Species consistency; (B) Genus consistency; (C) No consistency, consider synonyms of names | | | | | | |

**Up to harvest**

**At harvest**

**After harvest**

**Farm B**

Two holding dams

Irrigation pivot point

**Field:**
Produce
Soil

**Packhouse:**Produce transported in crates
Unwashed packed punnets*

**Processing facility:**
Unwashed packed punnets at receival
Crates at receival
Core wash water
Processing facility highcare:
Spinach after wash
Spinach after pack

Unwashed spinach punnets
Washed spinach packs

**Farm A**

River

Holding dam

Irrigation pivot point

**Field:**
Produce
Soil

**Packhouse:**
Floors; crates at receival; cutting surfaces; cutting machine; outside wash water bath; spinach bunches; RTE produce wash water; produce at receival, produce after cut
Packhouse highcare:
Produce after wash; produce after pack

Spinach bunches
RTE spinach pillow packs

**Farm C**

Holding dam

Irrigation pivot point

**Field:**
Produce
Soil

**Packhouse:**
Unwashed packed punnets
Crates in refrigerator (receival for washed products)

**Processing facility:**
Unwashed packed punnets at receival
Crates at receival
Core wash water
Processing facility highcare:
Spinach after wash
Spinach after pack

Unwashed spinach punnets
Washed spinach packs

Supplementary Figure S1: Different sampling points throughout the fresh produce supply chain selected for phenotypic antimicrobial resistance profile and genetic determinant ESBL/AmpC-producing Enterobacteriaceae analysis.
*punnets: plastic containers in which the baby spinach were packaged

**Retailer**

**Spinach production scenario 2**

**Spinach production scenario 1**
